# Fast-throughput simulations of laser-based additive manufacturing in metals to study the influence of processing parameters on mechanical properties

**DOI:** 10.1016/j.heliyon.2023.e23202

**Published:** 2023-12-10

**Authors:** Cameron McElfresh, Y. Morris Wang, Jaime Marian

**Affiliations:** a HRL Laboratories, Malibu, CA, 90265, USA; b Department of Materials Science and Engineering, University of California Los Angeles, Los Angeles, CA 90095, USA; c Department of Mechanical and Aerospace Engineering, University of California Los Angeles, Los Angeles, CA 90095, USA

**Keywords:** Laser additive manufacturing, Multiscale modeling, Crystal plasticity, Cellular automaton, Stainless steel, Copper, 316L, Hall-Petch effect

## Abstract

Laser-powder bed fusion additive manufacturing (LPBF-AM) of metals is rapidly becoming one of the most important materials processing pathways for next-generation metallic parts and components in a number of important applications. However, the large parametric space that characterizes laser-based LPBF-AM makes it challenging to understand what are the variables controlling the microstructural and mechanical property outcomes. Sensitivity studies based on direct LPBF-AM processing are costly and lengthy to conduct, and are subjected to the specifications and variability of each printer. Here we develop a fast-throughput numerical approach that simulates the LPBF-AM process using a cellular automaton model of dynamic solidification and grain growth. This is accompanied by a polycrystal plasticity model that captures grain boundary strengthening due to complex grain geometry and furnishes the stress-strain curves of the resulting microstructures. Our approach connects the processing stage with the mechanical testing stage, thus capturing the effect of processing variables such as the laser power, laser spot size, scan speed, and hatch width on the yield strength and tangent moduli of the processed materials. When applied to pure Cu and stainless 316L steel, we find that laser power and scan speed have the strongest influence on grain size in each material, respectively.

## 1 Introduction

Metal additive manufacturing (AM) is a rapidly growing technology that has revolutionized the way both consumer and high-performance products are designed and fabricated [1], [2], [3], [4], [5]. Metal AM, particularly laser power-bed fusion (LPBF), has enabled the rapid production of components with internal features, complex conformal cooling channels, and multi-material grading that enhance performance and would be difficult or impossible to fabricate using traditional manufacturing methods. Applications have been found across industries including aerospace [6], [7], biomedical [8], [9], and automotive [10], [11], among others. While AM is being adopted across many fields, uncertainties in the direct linkage between material properties and processing parameters remain a limitation for the rapid deployment of the technology. For instance, a lack of simple constitutive relationships that link printing parameters to the as-printed microstructure typically require that iterative experimental testing of printing parameters be performed for each newly printed material system to isolate the optimal settings [12], [13]. Further, the microstructure of additively manufactured parts can vary significantly depending on the processing variables, and, as such, the resulting mechanical and functional properties will reflect this variance as well [14], [15]. The chosen LPBF method (e.g., direct metal laser sintering or selective laser melting) can also significantly alter the outcomes of processed materials [16].

To reduce the overhead of iterative experimental testing, computational modeling is increasingly being used to simulate the microstructures resulting from LPBF processing [17], [18], [19], [20], [21], [22]. Models that take a physics-based approach to couple printing parameters (e.g., laser power, scan speed, hatch spacing, layer height) to solidification characteristics of the deposited material (e.g., specific heat, density, enthalpy, diffusion rates) can be utilized to predict features of the resulting microstructure, and in some cases, predict the mechanical behavior of the as-built components. However, high-fidelity modeling of the additive manufacturing process has proven difficult due to the influence of multi-scale and multi-physics phenomena such as nucleation and solidification, powder packing and multi-pass effects, fluid flow and Marangoni effects, martensitic transformations, as well as the contribution from defects such as key-holing, lack of fusion, vaporization, solute segregation, and hot cracking. As such, various simulation techniques have been employed to capture different mechanisms of the fabrication process, including phase field modeling (PFM) [23], [24], [25], [26], kinetic Monte Carlo (kMC) [27], [28], the finite element method (FEM) [21], [29], [30], [31], [32], [33], computational fluid dynamics (CFD) [20], [34], and cellular automata (CA) [35], [36], [37], [38], [39].

Despite these advances, an area still lacking investigation is the correlation between simulated advanced-manufactured microstructures and the calculation of their mechanical properties. AM microstructures typically consist of complex grain size distributions, textures, and, grain shapes, which are challenging to simulate considering direct energy deposition. Early efforts have focused on mapping digital image correlations of experimental microstructures with crystal plasticity (CP) simulations [40], [41]. In other works, PFM simulations have been coupled to CP simulations, integrating the microstructural evolution process with the mechanical analysis [42].

**Figure 1:**
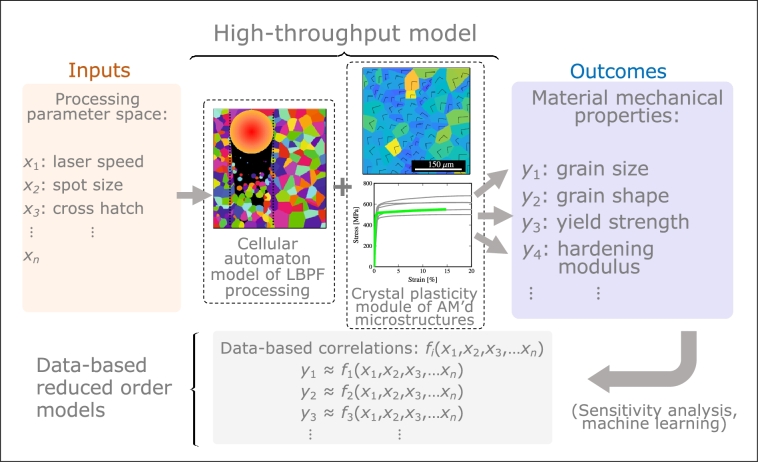
Schematic diagram of the approach developed in this paper, showcasing the relevant connections between input variables, mechanical property outcomes, and data-based surrogate models. The physics (high-fidelity) modules comprise a cellular automaton model for simulating LPBF processing and a crystal plasticity model for calculating the mechanical properties of the generated computational microstructures. The generally high computational overhead of these models leads to a high-fidelity data generating bottleneck, which may result in low quality data-based approximate models. Here, we focus on fast two-dimensional models for high throughput data generation and focus on data processing, machine learning, and sensitivity analysis.

Another aspect specifically related to LPBF that has not received sufficient attention is studying the effect of processing variables on the mechanical properties of the resulting microstructures [14]. The key variables in this case are the laser power, laser beam thickness, scan speed, and hatch spacing (i.e., the areal fraction of laser beam overlap during successive passes). In this work, we develop a CA model of dynamic melting and re-solidification in LPBF for pure Cu and 316L stainless steel as a function of the relevant laser processing variables. This is partially motivated by recent experimental studies that attempt to measure the influence of processing parameters on material properties [16]. Further, we post-process the resulting simulated microstructures using CP models that can capture complex polycrystalline size and shape features. Our study is intended to assess the relative importance of the processing parameters in relation to several selected mechanical properties of the AM structures. While three-dimensional models with fully coupled fluid dynamics, light-matter interaction, and non-equilibrium thermophysical phenomena have been developed, the intention of this work is to develop a fast approximate method to generate representative data for constitutive modeling reduced-order models. As such, this study is executed in two dimensions and areas for extension of the methods described here are discussed in Section 4.2. Fig. 1 shows a diagram with the workflow of our approach showcasing the relevant connections between input variables, mechanical property outcomes, and data-based surrogate models.

The paper is organized as follows: first, we provide a detailed description of the theory and methods used for the models (Section 2); we then carry out a systematic study of the effect of laser power, hatch spacing, scan speed, and laser spot thickness on the mechanical behavior of the resulting microstructures (Section 3); we follow in Section 4 with a discussion about the relevance of each parameter based on an exhaustive data analysis of the compiled results. We finalize the paper with our most important conclusions.

## 2 Theory and methods

Metal LPBF techniques achieve near-net part shaping by buildup of subsequently laser melted layers of powders in the 15∼45-μm range on an existing metal substrate [3], [43]. Simulating LPBF in this size range is extremely challenging, as it involves considering processes such as surface contact, friction, consolidation, and local melting, of which we have an incomplete understanding and can generally only model empirically [44], [45], [46]. Instead, as a surrogate model, in this work we consider fully-dense polycrystals with grains in the same size range as the elementary powders, acting both as the substrate and the fused layer. While this ignores a series of issues directly related to the granular nature of the powders, we consider it to be an acceptable approximation for this study despite its limitations, as will be discussed in Sec. 4.2.

### 2.1 Cellular automaton

The method employed in this work to simulate melting and solidification under the laser spot is a cellular automaton (CA) acting on a two-dimensional polycrystal discretized into a regular grid pattern [36], [37], [47]. Each cell in the grid is assigned an integer value that is either 0, corresponding to the cell being in the liquid state, −1 corresponding to the undercooled state, or >1 for solid cells belonging to a specific grain (indicated by the magnitude of the positive integer). Cells can change from the solid to liquid state both during melting, as described in Section 2.3, and during nucleation or growth, as described in Sections 2.4 and 3, respectively. For this study, a 300×300 μm region was discretized into a 1000×1000 grid. Table 1 contains a list of the different processes captured by the CA model.

**Table 1:** Rules for state change in the CA formulation. Note that $P_{\mathrm{nuclei}}=J(\overset{\to }{x})dx^{2}dt$ ($J(\overset{\to }{x})$ is the nucleation rate, defined in Sec. 2.4), *dx* and *dt* are, respectively, the size of the cell and the time step, *n* is the set of integer grain identifiers in the polycrystal, and *N* > 0 is a unique grain identifier.

State of Cell	Action	New State of Cell	Physical Description
*N* > 0	*T* > *T*_*m*_ in cell	−1	*S* → *L*
−1	*T* < *T*_*m*_ in cell	0	*L* → *L*_undercooled_
0	*P*_nuclei_ > *ξ* in cell	*N* ∉ *n*	*L* → *S*_nuclei_
0	Neighboring cell grows beyond *dx*	*n*_*i*_ ≡ identifier of neighboring cell	*L* → *S*_grain_

### 2.2 Polycrystal construction

To construct the two-dimensional polycrystal, we assign each point on the grid to grains according to a 2D Voronoi tessellation matching a log-normal distribution for a prescribed average grain size [48]. The grain distance is calculated using a scaled Euclidean distance metric:

(1) $$\mathrm{dist}\left(\overset{\to }{x^{1}},\overset{\to }{x^{2}}\right)={\left(\frac{\overset{\to }{x_{1}^{1}}-\overset{\to }{x_{1}^{2}}}{sx}\right)}^{2}+{\left(\frac{\overset{\to }{x_{2}^{1}}-\overset{\to }{x_{2}^{2}}}{sy}\right)}^{2}$$

where *sx* and *sy* are geometric scale factors to construct elongated grain microstructures and ${\overset{\to }{x}}_{1}$ and ${\overset{\to }{x}}_{2}$ are the coordinates of the grain centers. A microstructure with sx=1 and sy=1 has equiaxed grains while the selection of sx=1 and sy=0.5 generates quasi-elliptical grains elongated in the *y*-direction. Each grain, and by association all grid points associated with that grain, is assigned a random Euler orientation. In this study, all initial polycrystals were constructed with an equiaxed structure (i.e. sy=sx=1.0).

### 2.3 Laser temperature distribution

The local temperature distribution under the laser impingement spot is described using the Eagar-Tsai model [49], [50], which provides modified Gaussian profiles with elongated tails:

(2) $$T-T_{0}=\frac{\overset{˙}{P}}{\pi \rho C{\left(4\pi a\right)}^{1/2}}\int_{0}^{t}\frac{dt^{\prime }}{2a{\left(t-t^{\prime }\right)}^{3/2}}+\sigma^{2}\exp\left(-\frac{{\left(x-vt^{\prime }\right)}^{2}+y^{2}}{4a\left(t-t^{\prime }\right)+2\sigma^{2}}-\frac{z^{2}}{4a\left(t-t^{\prime }\right)}\right)$$

where $T_{0}$ is the initial temperature, $\overset{˙}{P}$ is the laser power, *ρ* is the mass density, *C* is the specific heat, *a* is the thermal diffusivity, *σ* is a beam radius distribution parameter, *v* is the speed of the beam, *t* is the time, and x,y,z are the spatial coordinates.

**Figure 2:**
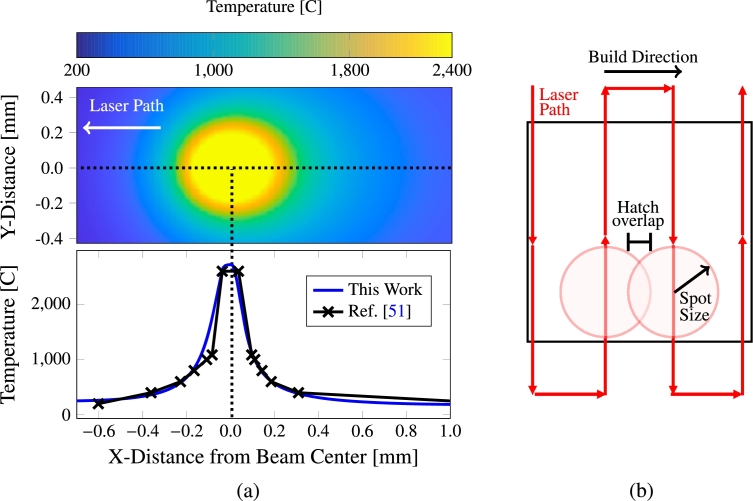
(a) Temperature distribution as a function of distance from the laser spot along the laser path, overlaid on experimental data from Ikeshoji et al. [51]. (b) Illustration of the anti-parallel scan pattern with two laser spots drawn on the laser path to denote the spot size and hatch overlap. The spot size is defined as the radius of the beam with *T* > *T*_*m*_ and the hatch spacing is defined as (hatch overlap)/(spot size).

The parameters in eq. (2) can be adjusted by comparing to carefully-conducted experiments of temperature profile evolution [51], [52]. Here we match the temperature profiles to those measured by Ikeshoji et al. [51] for pure Cu using a 800-W laser, as shown in Fig. 2(a). The laser spot follows a rastering pattern as shown in Fig. 2(b). As expected, the moving laser spot creates a non-uniform temperature distribution with a steeper thermal gradient ahead of the melt zone in the direction of motion and an elongated tail downstream of it, consistent with the Eagar-Tsai model. Matching eq. (2) to the experimental profiles in Fig. 2(a) leads to the values listed in Table 2 for the temperature profile models. The parameters for 316L are obtained using the same procedure based on results by Islam et al. [53], with the values also given on the table.

**Table 2:** Simulation parameters used during microstructure generation.

Parameter	Cu	316L	Units	Equation
*sx*	1.0	1.0	–	(1)
*sy*	1.0	1.0	–	(1)
*ρ*	8000	8960	kg⋅m^3^	(2)
*a*	3.5	117	mm^2^⋅s^−1^	(2)
*C*	770	385	J⋅kg^−1^⋅K^−1^	(2)
*σ*	0.05	0.05	mm	(2)
*T*_0_	273	273	K	(2)
*T*_*m*_	1101	1358	K	(3)
Δ*H*_*m*_	11.7	13.2	kJ⋅mol^−1^	(3)
*γ*^*SL*^	204	177	mJ⋅m^2^	(4)
*v*_0_	10^14^	10^14^	s^−1^⋅m^−3^	(5)
*Q*_*d*_	2.5	1.4	eV	(5)
*S*(*θ*)	0.5	0.5	–	(6)
*Q*_*g*_	2.94	2.19	eV	(7)

### 2.4 Nucleation and growth

As discussed in Sec. 2.1, initially, all points on the grid are assigned an integer value associated with a specific grain. As the laser beam sweeps across the sample surface, all cells immediately underneath it are assigned a temperature $T>T_{m}$ (close to 2500 ^∘^C, see Fig. 2, which is near the boiling pint of both Cu and 316L steel). As the laser beam continues on along the scan path, it leaves a pool of undercooled liquid where nucleation of a solid phase can occur. The free energy for nucleation due to an undercooling equal to Δ*T* is described in the standard form:

(3) $$\Delta G_{V}=\frac{\Delta H_{m}\Delta T}{T_{m}}$$

where $\Delta H_{m}$ is the latent heat of melting, $\Delta T=T-T_{m}$, and $T_{m}$ is the melting temperature. Assuming that surface energy, $\gamma^{sl}$, is the retarding force for the nucleation of spherical nuclei, the critical Gibb's free energy barrier for (homogeneous) nucleation can be written as:

(4) $$\Delta G_{\mathrm{hom}}^{⁎}=\frac{16\pi {\left(\gamma^{SL}\right)}^{3}}{3{\left(\Delta G_{V}\right)}^{2}}$$

The nucleation rate can then be expressed as a function of the frequency with which new atoms are added to a nucleus, *ν*, times the Boltzmann factor of the Gibb's energy barrier:

(5) $$J=\nu_{0}\exp\left(-\frac{\Delta G^{⁎}}{kT}\right)=\nu_{0}\exp\left(-\frac{Q_{d}}{kT}\right)\exp\left(-\frac{\Delta G^{⁎}}{kT}\right)$$

where $\nu_{0}$ is an attempt frequency, and $Q_{d}$ is the activation energy corresponding to the energy barrier of atoms attaching themselves to the new nuclei. However, during LPBF, nucleation is likely to take place heterogeneously, not directly within the melt pool but at existing grain boundaries, triple junctions, islands, partially formed nuclei, and other heterogeneous sites. Heterogeneous nucleation is introduced through the contribution of a shape factor that modifies the critical Gibb's free energy for homogeneous nucleation as:

(6) $$\Delta G^{⁎}=S(\theta )\Delta G_{\mathrm{hom}}^{⁎}$$

where S(θ)<1 and *θ* here represents the contact angle between a semi-spherical solid nucleus and an existing solid interface [54]. A given cell can engage in heterogeneous nucleation if the cell is in the liquid state and a neighboring cell is in the solid state.

**Figure 3:**
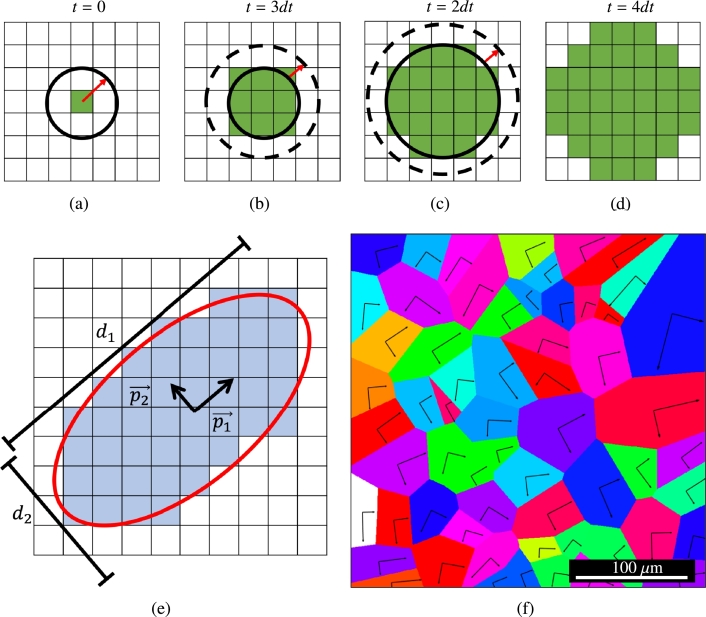
(a)-(d) Cellular growth of an unimpinged nuclei growing into neighboring cells from a central point. (e) Best fit of an ellipse (red) onto a single grain (blue) with the associated major and minor axes ($\overset{\to }{p_{1}}$ and $\overset{\to }{p_{2}}$, respectively) and dimensions (*d*_1_ and *d*_2_, respectively). (f) Polycrystal with the major and minor ellipsoid axes drawn as vectors originating from the center of the grain.

In terms of growth, in this work, we assume a planar growth regime such that a simple Arrhenius growth law can be used for the interface advancement of solid into the liquid. The growth velocity is given by the equation:

(7) $$v_{\mathrm{growth}}(T)=v_{0}\exp\left(-\frac{Q_{g}}{kT}\right)$$

where $v_{0}$ is a temperature-independent prefactor and $Q_{g}$ is the activation energy for interface propagation. For a growing grain or nucleus, the local temperature is taken as the average temperature integrated over the entire region in the grid that is occupied by the given unique grain identifier. Both nuclei and melt pool-adjacent grains are permitted to grow into the liquid at the same velocity. To account for radial symmetry in initially unimpinged growth of nuclei, new nuclei grow from their initial nucleation cell, (x,y), in a radial pattern until impinged by competing grains. The radius of a nucleated grain is then given by:

(8) $$r_{\mathrm{grain}}(t)=r_{\mathrm{grain}}(t-dt)+v_{\mathrm{growth}}dt$$

where $r_{\mathrm{grain}}(t-dt)$ is the grain radius at the previous time step and *dt* is the time step. This is exemplified in Fig. 3(a)-(d), which shows all the cells that satisfy $r_{\mathrm{grain}}(t-dt)>\mathrm{dist}({\overset{\to }{x}}_{\mathrm{center}}^{i},\overset{\to }{x})>r_{\mathrm{grain}}(t)$ and are transformed to *i* during *dt*. Here $\mathrm{dist}({\overset{\to }{x}}_{\mathrm{center}}^{i},\overset{\to }{x})$ is the Euclidean distance between the location of cell *i* and a given grain nucleation site, $\overset{\to }{x}$.

### 2.5 Time integration and coupling of the microstructure evolution to laser rastering

**Figure 4:**
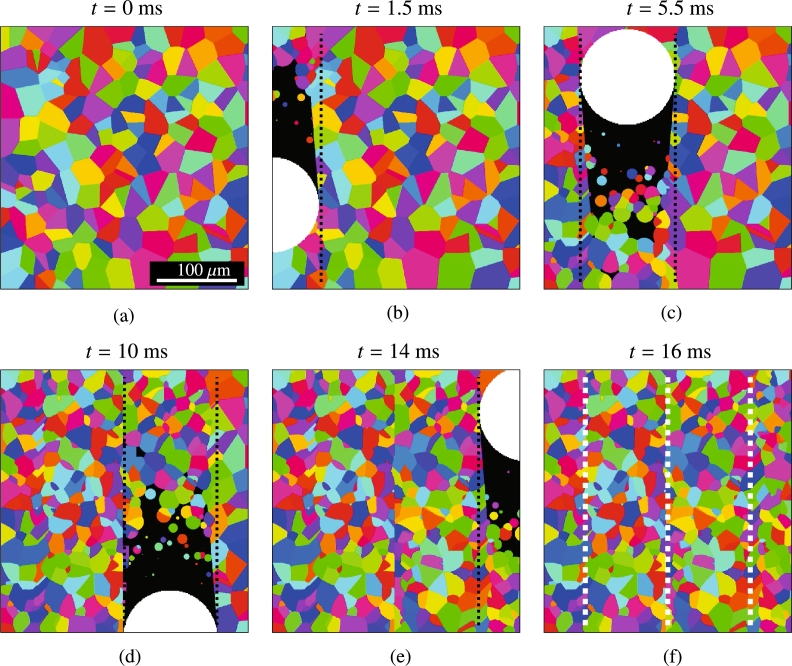
Evolution of the microstructure of a Cu substrate subjected to laser rastering with *v* = 0.1 m⋅s^−1^, *P* = 100 W, and 15% overlap. White: superheated liquid; black: undercooled liquid; color: different crystal orientations in solid grains. The dashed lines mark the edge of the laser spot in successive passes.

Prior to simulating the coupled system, the laser path and initial microstructure are constructed independently. The spatially dependent temperature distribution for each position on the beam path is pre-computed using the Eagar Tsai temperature profile described above. Every iteration, the beam is advanced in space and time and the local temperature distribution is updated accordingly, as shown in Fig. 4. A forward Euler time integration method is used to advance growing solid boundaries. The nucleation rate, $J(\overset{\to }{x})$ is calculated for each cell using eq. (5) and a new nucleus is formed at a given cell if $J(\overset{\to }{x})dx^{2}dt>\xi$, where *ξ* is a uniform random number in [0,1), *dx* is the size of each cell and *dt* is the time step. The time step was selected to be $dt=10^{-6}$ s. The remaining simulation parameters are given in Table 2.

Further, we adopt a directionally-parallel scan pattern, as shown in Fig. 2(b). In this work, we consider different degrees of overlap between successive passes, defined as the percentage of overlap relative to the laser spot size (the spot size is the radius of the beam, *ℓ*, where $T>T_{m}$), as shown in Fig. 2(b). The scan velocity is varied between 0.1 and 0.3 m⋅s^−1^, the laser power between 100 to 300 W, and the overlap between 25% and 75%. An example of microstructural evolution from a coupled simulation is shown in Fig. 4. Here the process was simulated with v=0.1 m⋅s^−1^, P=100 W, and 15% overlap between successive scan passes. The initial microstructure is shown in Fig. 4(a) with each grain being assigned a random orientation and colored accordingly. In Fig. 4(b)-(e) the beam moves across the sample with the liquid region (i.e., where $T>T_{m}$) shown in white while the undercooled liquid with $T<T_{m}$ is shown in black. New grains are observed to nucleate both in the melt pool and on the adjacent grain walls. Due to the high nucleation rate of grains in the melt pool trailing the beam spot (due to the high temperatures), an elongated ‘braid’-type microstructure is observed as is commonly observed in literature [3], [14], [55], [56], [57]. Lastly, the resulting microstructure is shown in Fig. 4(f). An animation of the process shown in the figure is included as supplementary information to this paper.

The next step in our model is to assess the mechanical ‘quality’ of the AM microstructures presented in the figure. For that, we choose to evaluate the yield strength and the hardening rate during deformation tests simulated using a CP model that accounts for arbitrarily shaped grains in 2D polycrystals. This is the subject of the next section.

### 2.6 Crystal plasticity model

The DAMASK code [58] was used to calculate the strength of the simulated microstructures. The DAMASK package uses an efficient spectral method to solve standard crystal plasticity problems using a number of different constitutive descriptions of dislocation-mediated crystal slip. Slip is computed on all available glide systems, defined by unit vectors ${\overset{\to }{n}}^{\alpha }$ and ${\overset{\to }{s}}^{\alpha }$, which represent the plane normal and shear direction in a given slip system *α*, respectively. For fcc metals, the slip rate can be defined as [40], [58], [59]:

(9) $${\overset{˙}{\gamma }}^{\alpha }={\overset{˙}{\gamma }}_{0}^{\alpha }{\left|\frac{\tau^{\alpha }}{g^{\alpha }}\right|}^{1/m}\mathrm{sign}\left(\tau^{\alpha }\right)$$

where ${\overset{˙}{\gamma }}_{0}^{\alpha }$ is a reference strain rate, $\tau^{\alpha }$ is the resolved shear stress (RSS), *m* is the strain-rate sensitivity (SRS) exponent, and $g^{\alpha }$ is the glide resistance in slip system *α* [59], [60]:

(10) $$g^{\alpha }=g_{0}+\mu b\left(\frac{c}{\lambda^{\alpha }}+\sqrt{\rho_{f}^{\alpha }}\right)$$

where $g_{0}$ is the intrinsic lattice resistance, *μ* is the shear modulus, *c* is a dimensionless parameter that captures the intensity of dislocation pileups, and *b* is the Burgers vector. Here, $\lambda^{\alpha }$ is a length scale set by the grain size and shape, and $\rho_{f}^{\alpha }$ is the *forest* dislocation density. Expressions for $\rho_{f}^{\alpha }$ as well as dislocation density evolution laws in metals can be found in our previous publications [48], [61], [62]. Other factors contributing to $g^{\alpha }$ such as solid solution strengthening and chemical effects can also be considered but are outside the scope of this work. For its part, $\lambda^{\alpha }$ is obtained as:

(11) $$\lambda^{\alpha }=\min_{\beta }\left\{{\overset{\to }{s}}^{\alpha }\cdot {\overset{\to }{d}}_{\beta }\right\}$$

where ${\overset{\to }{d}}_{\beta }$ (in 2D, β=1,2) are the principal axes of the *gyration* tensor representing a given grain. For a generic grain *i* enclosing a set of *N* spatial points $\left\{{\overset{\to }{r}}_{i}\right\}$ (typically, the mesh points contained within), the gyration tensor is defined as:

(12) $$\overset{\to }{R}=\frac{1}{N}\sum_{i}^{N}{\overset{\to }{r}}_{i}⊗{\overset{\to }{r}}_{i}$$

In this fashion, grains of arbitrary size are approximated as ellipsoids with an aspect ratio given by $d_{1}/d_{2}$ (where $d_{1}=\|{\overset{\to }{d}}_{1}\|$, $d_{2}=\|{\overset{\to }{d}}_{2}\|$), and $\lambda^{\alpha }$ is taken as the maximum of the projection of the slip direction ${\overset{\to }{s}}^{\alpha }$ with the two principal axes. An illustration of the method to calculate the principal axes, $d_{1}$ and $d_{2}$, in a square mesh, corresponding to the long and short radii of an ellipse in 2D, is given in Fig. 3(e). Each grain in the polycrystal is assigned the proper major and minor elliptical axes, as shown in Fig. 3(f).

One can also use shape descriptors ascribed to the gyration tensor to obtain useful information about each grain. For example, the grain size, $D_{i}$, can be obtained as the *radius of gyration*:

(13) $$D_{i}=\sqrt{{\left(d_{1}\right)}_{i}^{2}+{\left(d_{2}\right)}_{i}^{2}}$$

from which the average grain size in a polycrystal containing *M* grains, $\overset{¯}{D}$, can be obtained as:

(14) $$\overset{¯}{D}=\frac{1}{M}\sum_{j}D_{j}$$

Similarly, we can define the *aspect ratio*, $a_{i}={\left(d_{1}\right)}_{i}/{\left(d_{2}\right)}_{i}$, which is equal to one for equiaxed grains. Averaging over the entire grain population yields the mean aspect ratio, $\overset{¯}{a}$.

Equations (9)-(14) provides a ‘first principles’ approach to calculate GB strengthening and we have confirmed that it leads to a Hall-Petch dependence of the strength on $\overset{¯}{D}$. A systematic study varying the grain size of polycrystalline samples is performed in Appendix B, which returns a Hall-Petch dependence for the stress, thereby validating this approach. Thus, going forward, we take this as a valid procedure to calculate the strength of the AM structures. A complete list of the parameters used during the crystal plasticity simulations is given in Table 3. Additional forest hardening parameters for Cu and 316L were taken from the literature [63], [64].

**Table 3:** Parameters used in the crystal plasticity simulations. All other CP parameters were taken from ref. [73].

Parameter	316L Steel	Cu	Units	Source
*b*	0.254	0.26	nm	[65], [66]
*g*_0_	200	15	MPa	[58]
*m*	0.025	0.040	–	[67], [68]
*γ*_0_	0.09	0.001	s^−1^	[69], [70]
*C*_11_	207	170	GPa	[71], [72]
*C*_12_	135	122	GPa	[71], [72]
*C*_44_	130	76	GPa	[71], [72]
*μ*	79	88	GPa	[Appendix A]

### 2.7 Computational cost

All simulations were run on a 2019 MacBook Pro with a 1.4 GHz Quad-Core Intel i5 processor. No parallelization schemes were implemented for the CA modeling, though the adopted CA method is readily parallelizable [31], [38]. Native multi-threading was utilized for the DAMASK spectral solver. A total of 480 simulations were run (240 for Cu, 240 for 316L), requiring approximately 225 computing hours for combined CA and CP modeling, averaging 28.1 minutes per simulated microstructure. This is significantly less than finite-element method (2∼50 hours [33], [74], [75]) or phase field simulations (100s to 1000s of hours [24], [76]).

## 3 Results

### 3.1 Influence of processing parameters on microstructural properties

Next we explore the effect of the most relevant processing parameters on the properties of the resulting material microstructures. Here, we vary the laser power $\overset{˙}{P}$, laser spot speed *v*, and hatch spacing *h*, and study their effect on grain size $\overset{¯}{D}$, aspect ratio $\overset{¯}{a}$, tangent moduli (hardening rates, *H*), and yield strengths $\sigma_{y}$.

**Figure 5:**
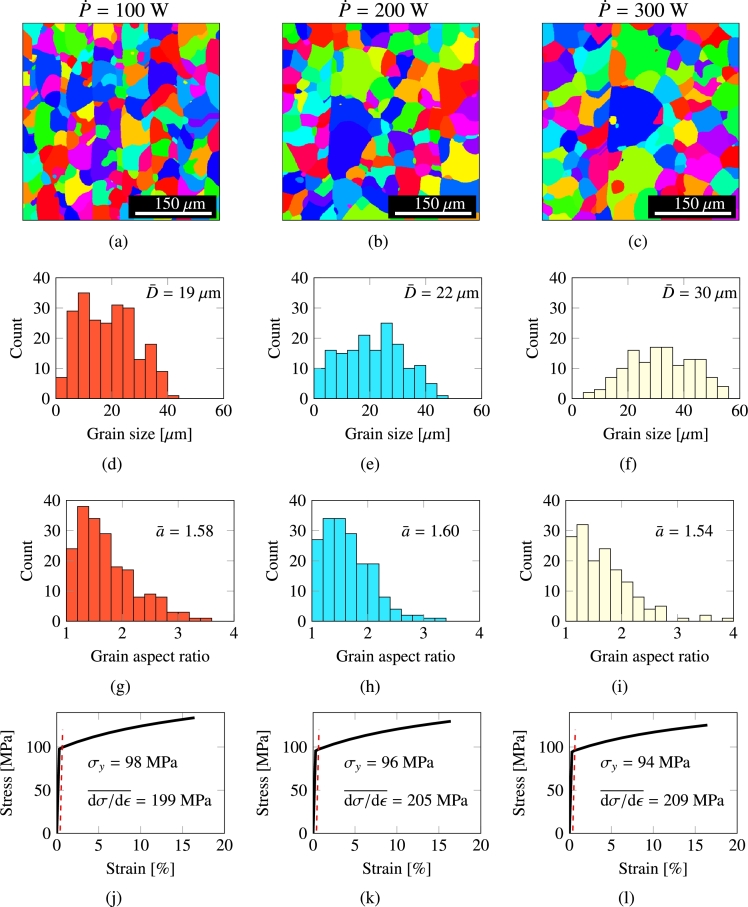
AM Microstructures of Cu for a fixed laser pass speed of *v* = 0.3 m⋅s and hatching of 20% using (a) $\overset{˙}{P}=100$ W, (b) 200 W, and (c) 300 W. The corresponding grain size distributions are shown in (d), (e), and (f). Aspect ratios in (g), (h), and (i), and stress-strain curves in (j), (k), and (l).

To illustrate the connections between processing parameters and microstructural properties, we first study the effect of the laser power on the grain size and the yield strength of Cu specimens for a fixed spot velocity of 0.3 m⋅s^−1^ and an areal overlap of 20%. We consider three values of $\overset{˙}{P}=100,\;200$ and 300 W. The results are shown in Fig. 5, where we first show in Figs. 5(a)-5(c) the resulting microstructures after the entire scan area has been processed by the laser. The associated grain size and asphericity distributions are given in Figs. 5(d)-5(f) and 5(g)-5(i), respectively, while the stress-strain curves obtained using the full polycrystal plasticity model are given in 5(j)-5(l). From simulations such as these, we can now extract $\overset{¯}{D}$, $\overset{¯}{a}$, *H*, and $\sigma_{y}$ for both materials systematically varying $\overset{˙}{P}$, *h*, and *v*. We present those results in the upcoming subsections.

### 3.2 Grain size and aspect ratio

#### 3.2.1 Results for pure Cu

**Figure 6:**
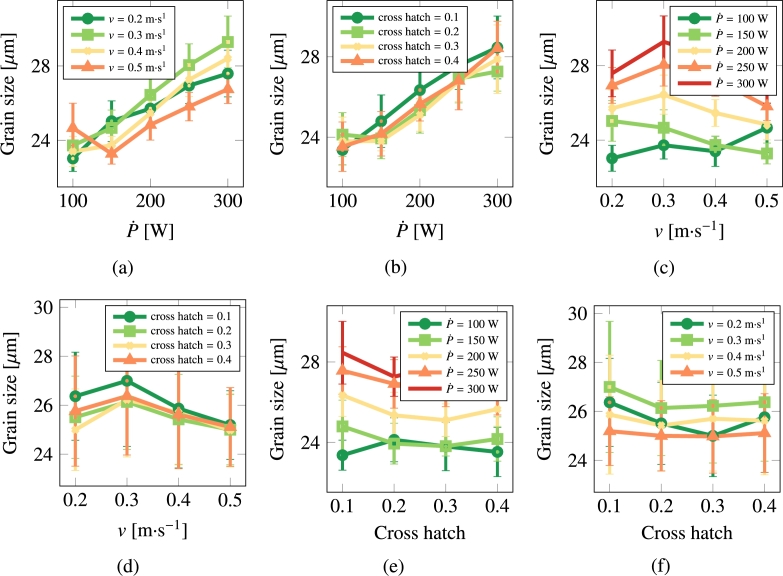
Grain size as a function of scan speed, laser power, and cross hatch for Cu.

**Figure 7:**
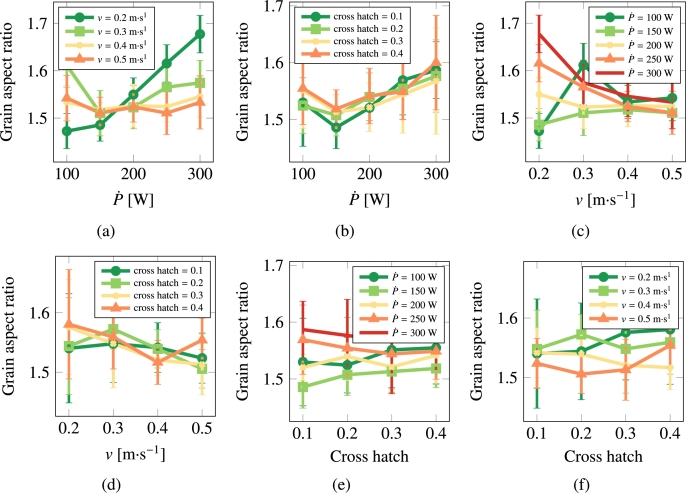
Grain asymmetry as a function of scan speed, laser power, and cross hatch for Cu.

Figs. 6(a)-6(f) and 7(a)-7(f) show the results for pure Cu. Visual inspection suggests that the dominant parameter in terms of the grain size and grain aspect ratio of the processed microstructures is the laser power $\overset{˙}{P}$. A quantitative correlative analysis is performed in Section 4.

#### 3.2.2 Results for pure 316L

**Figure 8:**
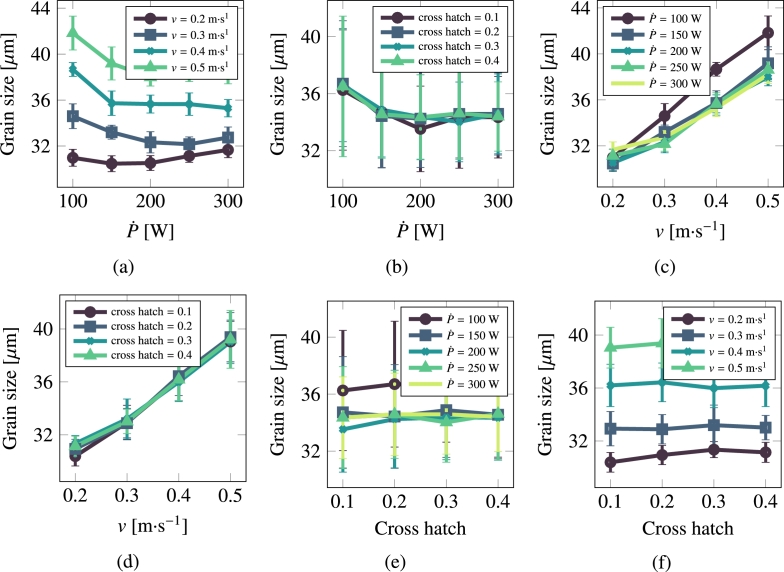
Grain size as a function of scan speed, laser power, and cross hatch for stainless steel 316L.

**Figure 9:**
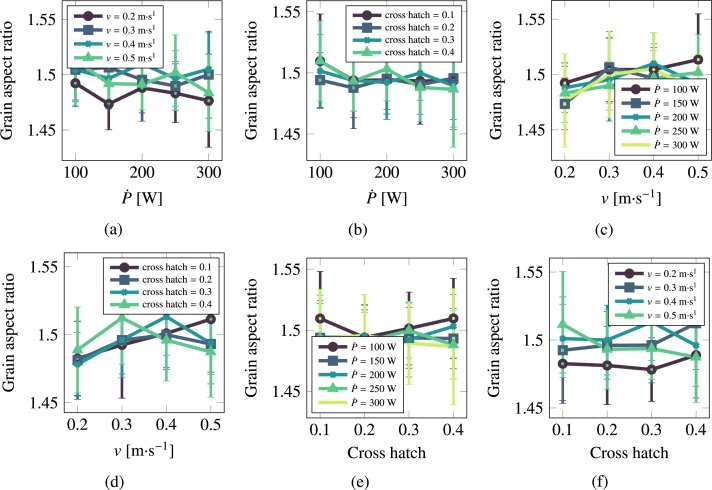
Grain asymmetry as a function of scan speed, laser power, and cross hatch for 316L.

Figs. 8(a)-8(f) and 9(a)-9(f) show the results for stainless steel 316L. Contrary to Cu, visual inspection in this case suggests that the dominant parameter is the laser scan speed, *v*. This will also be reflected in the quantitative correlative analysis presented in the discussion section.

### 3.3 Mechanical properties

#### 3.3.1 Results for pure stainless steel 316L

**Figure 10:**
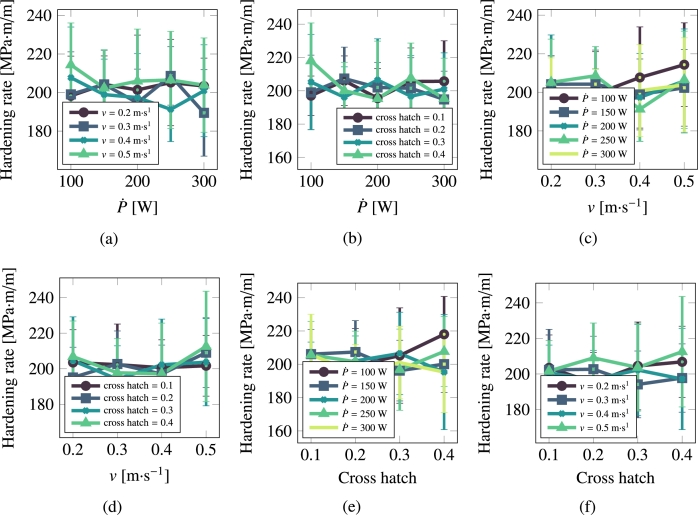
Hardening rate as a function of scan speed, laser power, and cross hatch for 316L.

**Figure 11:**
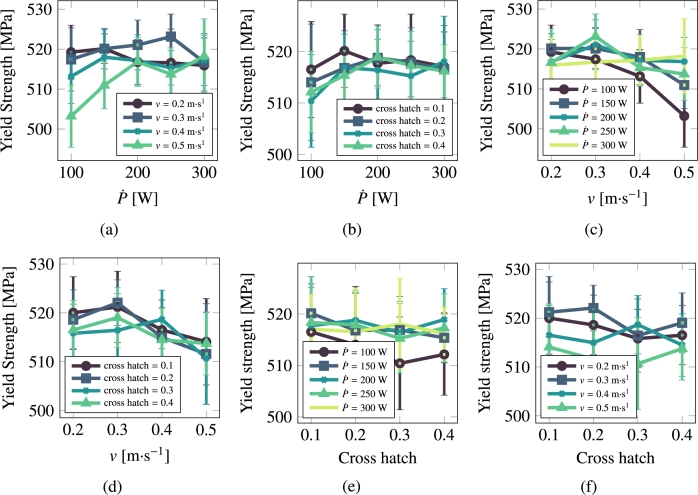
Yield strength as a function of scan speed, laser power, and cross hatch for 316L.

Figure 10, Figure 11 show the hardening rate and yield strength of stainless steel 316L as a function of processing parameters.

#### 3.3.2 Results for pure Cu

**Figure 12:**
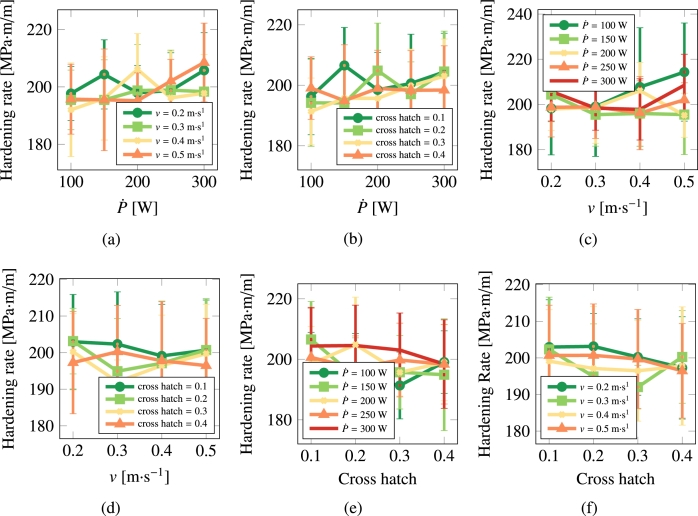
Hardening rate as a function of scan speed, laser power, and cross hatch for Cu.

**Figure 13:**
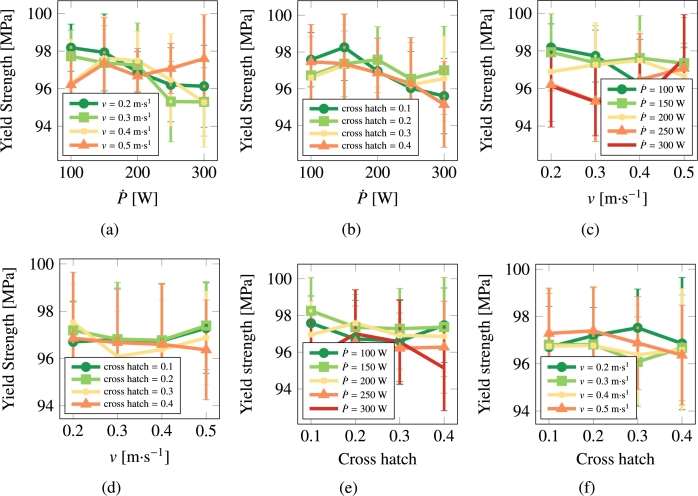
Yield strength as a function of scan speed, laser power, and cross hatch for Cu.

Figure 12, Figure 13 show the hardening rate and yield strength of pure Cu as a function of processing parameters.

### 3.4 Statistical importance analysis

Given the multidimensional nature of the problem under study and the large amount of data presented in the previous sections, it is important to establish correlations between the processing parameters and the material property outcomes. Next we perform a multivariate fitting to assess the importance of each AM processing parameter by fitting to an expression of the type:

(15) $$\mathrm{Property}=A{\left[\overset{˙}{P}\right]}^{n_{1}}+B{\left[v\right]}^{n_{2}}+C{\left[h\right]}^{n_{3}}+D$$

where *A*, *B*, and *C* are fitting constants that indicate the degree and sign of the correlation. *D* is an independent constant that reflects the intrinsic importance of each variable. $n_{1}$, $n_{2}$, and $n_{3}$ are fitting exponents, although in view of the results presented above, we simply set them to unity in this study. To capture the true importance of each parameter, i.e., removing the effect of the absolute values, which vary significantly across $\overset{˙}{P}$, *v* and *h*, we normalize each one to variables defined in the interval [0:1]. *h* (cross hatch) is naturally defined that way, so it does not require any normalization. For $\overset{˙}{P}$ and *v*, we simply do:

$$\left[x_{i}\right]=\frac{x_{i}-x_{\min}}{x_{\max}-x_{\min}}$$

Here we use ${\overset{˙}{P}}_{\max}=300$ W and $v_{\max}=0.5$ m⋅s^−1^. The lower limit for both parameters is taken as zero. The above values reveal two interesting facts. First, the only relevant processing parameter as it relates to microstructural properties in Cu is the laser power, which, with values of A=0.615 and 0.225, is the dominant factor in determining the grain size and grain aspect ratio, respectively. Second, except for the two said correlations of $\overset{¯}{D}$ and $\overset{¯}{a}$ with $\overset{˙}{P}$, the independent constant, *D*, representing an intrinsic baseline value of each property is the most influential factor in the microstructural properties of Cu. As it relates to steel, the main correlation is that of the laser scan speed with the grain size (B=0.904) and with the yield strength (inverse: B=−0.223), with the rest showing only marginal or negligible correlation.

It is also of interest to perform a direct fitting, where normalization is not used for the processing parameters or the response variables. The coefficients A,B,C, and *D* are added to Table 4 in parentheses. *A*, *B*, and *C* are understood to have units of property per power unit, property per velocity, and plain property, respectively, while *D* is given in units of the property in question (μm for grain size, m/m for grain asymmetry, MPa for the yield strength, and MPa⋅m/m for the hardening rates).

**Table 4:** Best fit parameters for the normalized response for pure Cu and 316L stainless steel. Response variables (grain size, grain asymmetry, yield strength, hardening rate) were normalized using the min-max scheme: $x_{\mathrm{norm}}^{i}=\left(x^{i}-\min(x)\right)/\left(\max(x)-\min(x)\right)$. The numbers in parentheses correspond to a direct (non-normalized) fitting.

Material	Property	$A\;\left(\overset{˙}{P}\right)$	*B* (*v*)	*C* (*h*)	*D*
Cu	Grain size	(5.3)	−**0.185** (−1.6)	−**0.064** (−0.55)	**0.111** (19.7 [μm])
	Grain asymmetry	**0.225** (0.08)	−**0.191** (−0.07)	**0.047** (0.02)	**0.357** (1.53 [μm/μm])
	Yield strength	−**0.192** (−2.14)	−**0.022** (−0.25)	−**0.050** (−0.50)	**0.695** (98.72 [MPa])
	Hardening rate	**0.109** (8.90)	−**0.034** (−2.80)	−**0.064** (−5.24)	**0.510** (198.01 [MPa⋅m/m])

316L	Grain size	−**0.174** (−1.29)	(6.97)	−**0.167** (0.15)	**0.019** (13.31 [μm])
	Grain asymmetry	−**0.078** (0.01)	**0.121** (0.02)	**0.003** (0.00)	**0.394** (1.49 [μm/μm])
	Yield strength	**0.101** (4.32)	−**0.223** (−9.58)	−**0.069** (−2.98)	**0.692** (522.22 [MPa])
	Hardening rate	−**0.046** (−6.30)	**0.045** (6.15)	**0.013** (1.74)	**0.396** (200.68 [MPa⋅m/m])

Our results can be qualitatively compared with experimental studies, such as that by Obeidi et al. [16], who in Table 4 of their paper calculate the level of correlation and its significance (confidence in the correlation based on the scatter in the data) for a number of material properties using different printing devices. Of those, only the ultimate tensile strength (UTS), and its relationship with $\overset{˙}{P}$ and *v*, is relevant for our study. All the correlation factors of the UTS with laser power were weak (and generally negative), and displayed a very low level of confidence. Its dependence with the scan speed showed a higher degree of (negative) correlation with improved confidence. This is in agreement with our results in Table 4 (where B=−0.223), although it must be said that the range of laser powers and scan speeds explored there was 160∼190 W and 0.8∼1.2 m/s, respectively.

**Figure 14:**
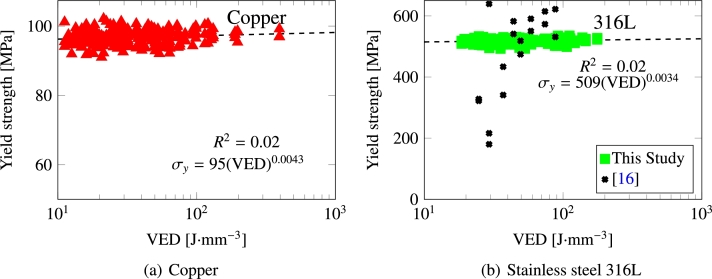
Yield strength as a function of volumetric energy density for (a) Cu and (b) 316L. Experimental results from ref. [16] are shown for steel.

An alternative approach is to consider a correlation variable that reflects the volumetric energy density (VED) of the process [53]:

(16) $$\mathrm{VED}=\frac{\overset{˙}{P}}{2v\ell^{2}h}$$

where 2*ℓ* is the laser spot size and (hℓ) is the hatch spacing. The correlation of the yield strengths of Cu and 316L steel with the VED from 240 independent simulations is given in Fig. 14. Fitting to a power law scaling of the type: $\sigma_{y}=E{\left(\mathrm{VED}\right)}^{n}$ yields the results shown in the figure, with the fitting constant *E* effectively taking the same values as the constant *D* in eq. (15) above. The correlation is weak, as illustrated by values of the exponents *n* close to zero in both cases. Thus, in terms of yield strength, the intrinsic values of the starting material do not change significantly on average in the processed materials. Also, it can also be seen that the scatter is small, with $R^{2}$ noted in both plots, so that one might not expect a significant degree of variability on either side of the average correlation. This is in contrast with the experimental results shown in Fig. 14(b), which display a much stronger dependence with the VED indicator [16]. This is not surprising given that Obeidi et al.'s experiments pertain to laser powder-bed fusion processing, whereas ours start from fully formed polycrystals. However, notwithstanding these differences, the agreement between the calculated and measured values of $\sigma_{y}$ in the 40 < VED < 100 range is worth noting.

## 4 Discussion

### 4.1 Discussion on evolution of microstructural and mechanical variables

It is interesting to comment on the evolution of some of the microstructural descriptors in both materials on the basis of the importance analysis performed in the previous section. In general, one can reduce the effect of the processing variables to the following considerations:

• As indicated by eq. (2), the laser power $\overset{˙}{P}$ provides the level of superheating in the liquid zone. The more superheating (i.e., the larger the laser power), the higher the driving force (as dictated by eq. (3)) for crystallization in the wake of the moving laser beam.
• The scan speed *v* has the effect of creating compressed and extended Gaussian thermal profiles ahead of and behind the laser spot, respectively. The faster the scan speed, the more extended the trailing thermal profile.
• The cross-hatch remelts an area of the material previously crystallized. However, if the overlap area has thermalized before the next passing of the laser beam (i.e., if all the heat deposited by the laser in that particular spot has dissipated), then this parameter has a very limited effect. In light of the results presented in Figure 6, Figure 7, Figure 8, Figure 9, this indeed appears to be the case in our study.

Eliminating the cross-hatch as a relevant parameter, we can now cross-compare the values of $\overset{˙}{P}$ and *v* with the nucleation and growth rates. In this study we do not solve the heat equation explicitly (instead we apply the Eagar-Tsai model assuming the thermal profile is in instantaneous equilibrium with the moving laser beam), and thus the effect of $\overset{˙}{P}$ is to establish the amount of superheating while the effect of *v* is to dictate the time over which that superheating remains for each spatial point. If *v* is high, spatial points in the wake of the moving laser beam thermalize quickly, diminishing the possibility of nucleation and growth. For this reason, it is difficult to separate the effects of these two as we have done in the correlations considered in eq. (15). In the case of Cu, it is $\overset{˙}{P}$ that has a bigger impact on the final average grain size, while in the case of 316L, it is *v*.

Other interesting observations include the fact that the aspect ratio in Cu is seen to always range between 1.5 and 1.7 regardless of the value of the processing parameters. We do not believe that this means that $\overset{¯}{a}$ is insensitive to a moving laser spot dragging a molten area with it, but simply that perhaps above a threshold scan speed and/or laser power, the aspect ratio converges to 1.5∼1.7. This is probably related to the fact that (i) the values of $\overset{˙}{P}$ considered here are always sufficiently high produce a high level of superheating, and (ii) that the maximum scan speed tried here is slower than the nucleation rate in the molten zone. Likewise, for steel, $\overset{¯}{a}$ stays at 1.5 regardless of the values of the processing parameters.

### 4.2 Discussion on model limitations

The main advantage of our methodology is that it captures both processing (i.e., re-solidification within the melt pool under a moving laser spot) and mechanical testing (through yield strength and hardening calculations) within a single framework. We have developed tools that seamlessly integrate microstructural evolution (using a model based on a cellular automaton) with polycrystal plasticity modeling, allowing us to directly connect simulated additively-manufactured microstructures with their mechanical properties. One of the most important aspects controlling the strength of the processed structures is the hardening due to grain boundaries in microstructures defined by complex grain shapes and wide size distributions. We have addressed this by developing a novel approach by which each grain is represented by the inscribed ellipsoid defined by the principal directions of the gyration tensor. Such approach makes it then straightforward to numerically extract individual grain sizes, aspect ratios, and the average grain diameter of the system. More importantly, we calculate the mean free path of dislocations in each grain by projecting these principal axes on the specific slip directions contained in it, which is then used to obtain the maximum characteristic size of dislocation sources. We have shown that this approach captures the fundamental features that lead to a macroscopic Hall-Petch strengthening response. As well, while cellular automata are more restricted in what can be simulated than other more general methods such as the phase field model, they provide a computational expediency that makes them ideal to explore a broad parametric space efficiently.

In terms of limitations, we can acknowledge some numerical and some physical. First, the current model, is limited to two dimensions, which, while suitable for special cases such as thin metal films (wafers) or under columnar grain substrates, limits its application to more general situations. A related point is that the model condenses the powder layer and the metal substrate into a single 2D polycrystal, which overlooks the granular nature of the powder layer and the defects that come with it, such as lack of fusion [77], [78], hot cracking [79], [80], [81], [82], keyhole pores [79], [83], balling [79], [84], and evaporation. Incorporating flaws into the models will be a crucial next step to account for realistic correlations between LPBF microstructures and their mechanical properties [85], [86], [87].

The selection of the Eagar-Tsai model to estimate the evolution of the temperature field provides a fast surrogate model for the evolving melt pool, but is admittedly limited in its assumptions to capture detailed thermophysical effects. Modified versions of the original point-wise moving heat source model [88] exist to account for different features of the melt pool, such as temperature dependent properties [89] and phase change effects [90]. An alternative approach to the modified analytical solutions involves solving the temperature field directly on a finite difference/element/volume mesh.

Considering 2D polycrystals also negates the possibility to correlate the texture of the underlying substrate with the solidified grains, as has been shown in multiple experiments [14]. Indeed, it has been shown that the properties of 316L steel may be controlled by the lengthscale of solidification cells [3], [5], [91], [92], [93], which grow in ‘colonies’ that preferentially follow crystal orientations dictated by the grains of the underlying substrate [14], [94], [95]. This will also be the subject of future improvements to the model, mainly by extension to 3D and by modifying the heterogeneous nucleation criterion (see eq. (6)) to capture the grain orientations of the substrate, as well as the commonly observed preferential 〈100〉 growth texture in cubic crystals, which is commonly incorporated using the de-centered octahedron algorithm [96], [97], [98]. Finally, an improved nucleation condition would consider heterogeneous nucleation on the melt pool's ‘diffuse’ boundary and potentially from non-molten grains inside the melt pool.

## 5 Conclusions

We conclude with our main findings, summarized below:

1. We have developed a suite of simulation tools to study the microstructural evolution of metallic material surfaces by laser-based processing and the connection of the resulting microstructure to the mechanical properties of the material.
2. A cellular automaton (CA) is first used to simulate the passing of a laser spot of fixed size over a pre-existing two dimensional polycrystal. The CA model captures melting and re-solidification with full spatial temperature dependence based on the Eagar-Tsai model, and can be parametrically used to change the laser power, scan speed, and hatch fraction (laser spot overlap).
3. We have implemented a new method to calculate grain boundary strengthening directly from intrinsic dislocation properties and grain geometry, without assuming a Hall-Petch relationship. Our method is based on restricting the mean free path of dislocations by the shortest principal axis of the ellipsoid inscribed in the grain. Our approach yields a Hall-Petch dependence on average grain size when applied to polycrystals.
4. The grain boundary strengthening model is implemented in a polycrystal plasticity framework from which the yield strength and hardening rates (tangent modulus) can be extracted. We apply the full model to pure Cu and to stainless steel 316L.
5. We have studied the correlation strength of the three processing variables, laser power, laser scan speed, and cross-hatch, with two microstructural properties, grain size and grain aspect ratio, and two mechanical properties, the yield strength and the hardening rate.
6. We find the strongest correlation for pure Cu for the [grain size]-[laser power] pair, while for 316L it is [grain size]-[scan speed]. We also find that the cross-hatch has practically no influence on the microstructural properties of Cu or 316L.

## CRediT authorship contribution statement

**Cameron McElfresh:** Writing – original draft, Visualization, Software, Methodology, Investigation, Formal analysis, Conceptualization. **Y. Morris Wang:** Writing – review & editing, Validation, Funding acquisition, Conceptualization. **Jaime Marian:** Writing – original draft, Supervision, Methodology, Investigation, Funding acquisition, Formal analysis, Conceptualization.

## Declaration of Competing Interest

The authors declare that they have no known competing financial interests or personal relationships that could have appeared to influence the work reported in this paper.

## Figures and Tables

**Table:** 

Parameter	316L Steel	Cu
*μ*_V_	92	55
*μ*_R_	67	122
*μ*	79	88

**Figure B.15:**
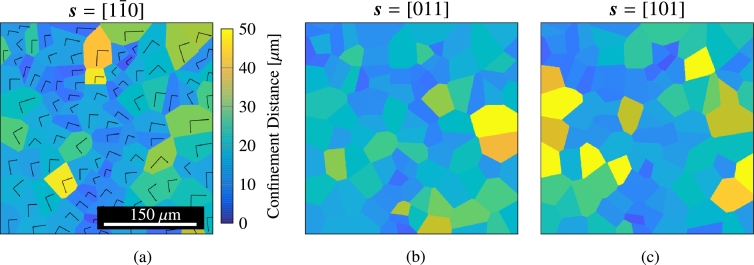
The grain boundary confinement in the (b) [101], (c) [011], and (a) $\left[1\overset{¯}{1}0\right]$ directions. Each grain's major and minor elliptical axis is marked with a vector on plot (a).

**Figure B.16:**
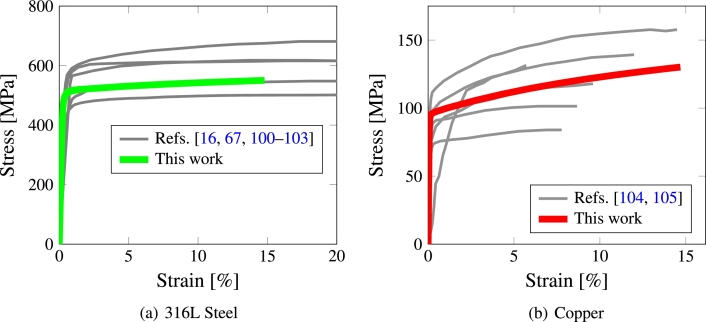
Comparison of CP calculations using the model described in Sec. 2.6 and several related experimental results. Engineering stress-strain curves for (a) 316L [16], [67], [100], [101], [102], [103] steel and (b) Cu [104], [105].

**Figure B.17:**
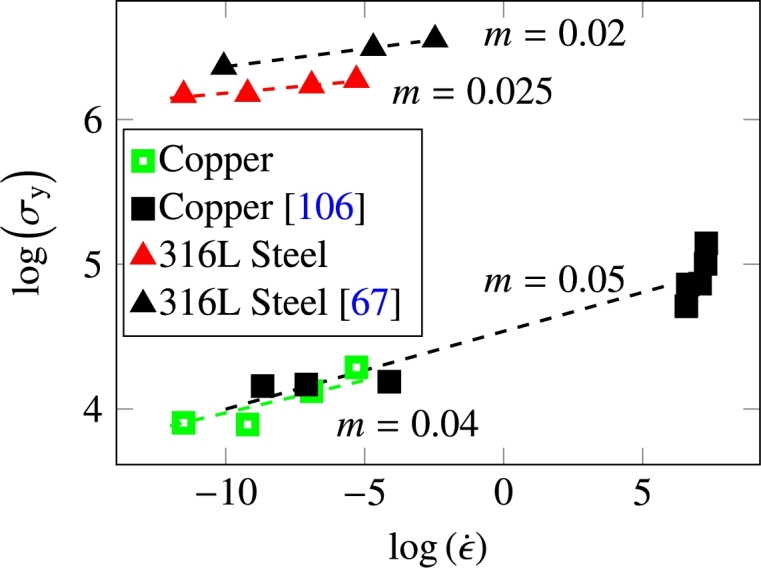
Log yield strength versus log strain-rate plot to extract the strain rate sensitivity for the simulated Cu and 316L steel plotted alongside experimental data from [67], [106]. The simulated microstructures consist of 100 grains in a 300 × 300-μm grid. The strain rate sensitivity constant, $m=\Delta \ln(\sigma_{y})/\Delta \ln(\overset{˙}{\epsilon })$ is given for each set of data.

**Figure B.18:**
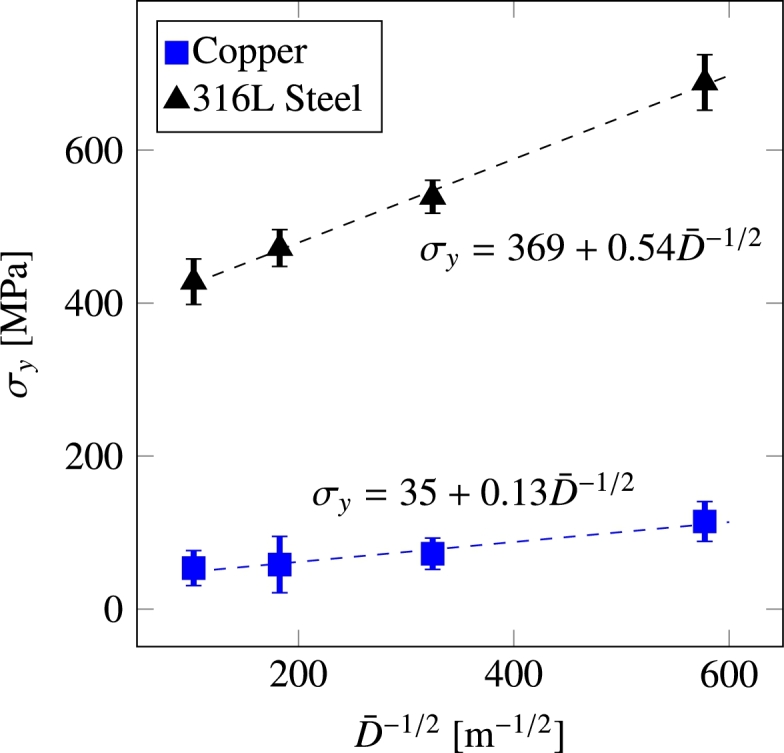
Variation of the yield strength with average grain size (plotted using the Hall-Petch scaling) for 316L steel and pure Cu using the crystal plasticity model introduced in Section 2.6. Hall-Petch fits to the data give the ground strength (lattice friction) and the Hall-Petch constant. Results are given in units of MPa. The error bars represent the variation on the results from simulating three different microstructures at the given conditions.

## Data Availability

Data associated with this study have not been deposited in a public repository. However, they can be made available upon reasonable request to the corresponding author(s) of this paper.

## Appendix A Anisotropic elastic constants

The shear modulus is calculated using the average of the Voight and Reuss definitions [99]:

(A.1) $$\mu_{V}=\frac{1}{5}\left(C_{11}-C_{12}+3C_{44}\right)$$

(A.2) $$\mu_{R}=\frac{15}{4\left(S_{11}-S_{12}\right)+3S_{44}}$$

(A.3) $$\mu =\frac{1}{2}\left(\mu_{V}+\mu_{R}\right)$$

where,

(A.4) $$S_{11}=\frac{C_{11}+C_{12}}{\left(C_{11}-C_{12}\right)\left(C_{11}+2C_{12}\right)}$$

(A.5) $$S_{12}=-\frac{C_{12}}{\left(C_{11}-C_{12}\right)\left(C_{11}+2C_{12}\right)}$$

(A.6) $$S_{44}=\frac{1}{C_{44}}$$

Using the values in Table 3 for $C_{ij}$, we get:

all values in GPa.

## Appendix B Validation of dislocation mean free path approach for polycrystal strength calculations

Here we carry out simulations of polycrystal strength using the crystal plasticity approach described in Sec. 2.6. The objective is to validate the model by comparing against measurements published in the literature in materials with well-controlled microstructures so that the model can then be confidently applied to AM microstructures.

### B.1 Dislocation mean free path

First, using the approach described in Sec. 2.6, we map the principal axes of  (from eq. (12)) for each grain belonging to a generic polycrystal with a log-normal grain size distribution. We then generate a color map of the same polycrystal to illustrate the value of $\lambda^{\alpha }$ according to eq. (11). We study three slip directions, $\overset{\to }{s}=[1\overset{¯}{1}0],\;[011],\;[101]$, all in the (110) plane. The results are shown in Figs. B.15(a)-(c). Except for a few outliers with clearly larger values of $\lambda^{\alpha }$, which display yellow tonalities, most grains display similar values of the principal axes, consistent with the use of a log-normal distribution (equiaxed) to generate the polycrystals. Grains with large $\lambda^{\alpha }$ are expected to be softer, which may lead to localization if there is not a sufficient number of them to allow the material to deform uniformly. We emphasize that the principal axes shown for each grain in Fig. B.15(a) refer to the size of the grains, not its crystal orientation.

### B.2 Simulations of grain boundary strengthening

Next, we show stress-strain curves from uniaxial tension tests of a 316L and Cu polycrystals with an approximate grain size of $\overset{¯}{D}=40$ μm in Figs. B.16(a) and B.16(b), respectively, along with experimental results taken from literature. We find good agreement between our simulated results and experimentally determined values, which gives us confidence to then use the model to calculate the strength as a function of grain size.

Another useful check is that of the dependence of the strength with strain rate, from which the strain-rate sensitivity exponent (SRS) can be extracted. The SRS exponent is prescribed in our CP model (see Table 3), and so a self-consistency check is to run CP simulations at different strain rates and then obtain the corresponding yield strengths. Fig. B.17 shows results for both 316L steel and pure Cu and a comparison top the relevant experiments. As the results show, the SRS exponent extracted is consistent with that used in eq. (9), adding confidence to the model.

To obtain the strengthening to be ascribed to grain boundaries, we systematically vary the average grain size in the polycrystals and calculate the yield strength as a function of ${\overset{¯}{D}}^{-1/2}$. The results are plotted in Fig. B.18.

## Appendix C Supplementary material

Video Simultaneous animation of the temperature field and nucleation and growth of grains during laser spot motion using the models developed in this paper. Video
